# SharpGAN: Dynamic Scene Deblurring Method for Smart Ship Based on Receptive Field Block and Generative Adversarial Networks

**DOI:** 10.3390/s21113641

**Published:** 2021-05-24

**Authors:** Hui Feng, Jundong Guo, Haixiang Xu, Shuzhi Sam Ge

**Affiliations:** 1 Key Laboratory of High Performance Ship Technology, Wuhan University of Technology, Ministry of Education, Wuhan 430063, China; feng@whut.edu.cn (H.F.); jundong@whut.edu.cn (J.G.); 2 School of Transportation, Wuhan University of Technology, Wuhan 430063, China; 3 Department of Electrical & Computer Engineering, National University of Singapore, Singapore 117576, Singapore; samge@nus.edu.sg

**Keywords:** image deblurring, object detection, smart ship, GAN

## Abstract

Complex marine environment has an adverse effect on the object detection algorithm based on the vision sensor for the smart ship sailing at sea. In order to eliminate the motion blur in the images during the navigation of the smart ship and ensure safety, we propose SharpGAN, a new image deblurring method based on the generative adversarial network (GAN). First of all, we introduce the receptive field block net (RFBNet) to the deblurring network to enhance the network’s ability to extract blurred image features. Secondly, we propose a feature loss that combines different levels of image features to guide the network to perform higher-quality deblurring and improve the feature similarity between the restored images and the sharp images. Besides, we use the lightweight RFB-s module to significantly improve the real-time performance of the deblurring network. Compared with the existing deblurring methods, the proposed method not only has better deblurring performance in subjective visual effects and objective evaluation criteria, but also has higher deblurring efficiency. Finally, the experimental results reveal that the SharpGAN has a high correlation with the deblurring methods based on the physical model.

## 1. Introduction

Smart ships use the equipped sensors to percept the surrounding environment (for example, obstacles, such as ships, buoys, etc.) without the help of sailors. The perception information is analyzed and processed through the computer and control system to make recommendations for ship’s navigation decisions. In addition, smart ships also need to realize autonomous navigation based on planned navigation routes in different sailing scenarios and complex environmental conditions such as open waters, narrow waterways, entering and leaving ports, and docking and leaving docks.

Smart ships can be divided into three modules: the perception module, the decision-making module, and the control module. The perception module provides obstacles and ship motion information for the decision-making module by perceiving the ship’s motion state and surrounding environment. The decision-making module uses the information provided by the perception module to plan a safe route for the ship’s navigation. The control module controls the course and speed of the ship according to the route planned by the decision-making module.

In the perception module, the visual perception based on visible light is the core of the perception technology of smart ships. Vision sensors (such as cameras) are significant sensors for detecting and identifying the obstacles. However, the performance of visual sensors to identify obstacles is obviously degraded by the motion of ship, especially if the ship is affected by wind, wave, and current, which leads to motion blur of the objects in the image and weakens the objects’ feature. This will reduce the accuracy of the visible light-based obstacle object detection algorithm, which makes it difficult to provide accurate obstacle avoidance information for the subsequent decision-making module, thereby reducing the safety performance of smart ships. Furthermore, the ship running on the water is very different from the car running on the land. Due to the large inertia and small damping, its movement flexibility is very poor, so it is necessary to detect the obstacle information in advance as far as possible. Therefore, in order to ensure the navigation safety of smart ships, effectively removing motion blur from the images is of great significance.

Since the image deblurring problem is ill-posed, the traditional deblurring methods [1,2,3,4,5,6,7,8,9] set up some constraints (such as uniform blur/non-uniform blur) and combine the prior information of the blurred images to establish a corresponding mathematical model to solve the latent sharp images. These methods have very high computational complexity, and the deblurring effect of the models also has great dependence on the setting of model parameters. In addition, since the establishment of the models relies on the corresponding constraints, these methods are difficult to adapt to the actual deblurring problems. With the rapid development of deep learning in recent years, many image deblurring methods based on convolutional neural networks and generative adversarial networks have been proposed [10,11,12,13,14]. Most of these methods improve the network’s ability to extract image features by stacking a large number of convolutional layers or ResBlocks (residual blocks) [15], while obtaining a larger range of receptive fields to improve the quality of restored images. These methods have achieved good deblurring effects, but the ability of simply stacked convolutional layers to extract image features is not sufficient. Secondly, although it is important to improve the clarity of blurred images, it is also necessary to ensure that restored images, and sharp images have highly similar image features. The existing deep-learning-based image deblurring method [14] uses the conv_3-3 layer of the VGG19 (Visual Geometry Group) network [16] to construct the content loss function to improve the feature similarity between the restored images and the sharp images, but this does not make full use of the powerful image feature extraction ability of the VGG19 network. Moreover, in actual navigation, the obstacles around the ship are dynamically updated. New obstacles need to be identified and avoided in time to prevent collisions between ships and obstacles. Smart ships, especially the small-sized unmanned surface vehicles (USVs), have limited computing power, which puts forward higher requirements for the lightweight and real-time performance of the deblurring algorithm in the perception module. Therefore, a good image deblurring algorithm for smart ships needs to balance the accuracy and real-time performance of deblurring. However, the existing deblurring algorithms are difficult to make a trade-off between them. Many algorithms even need ten seconds or more to deblur a single image [6,7,9,10,13], which greatly affects the real-time obstacle avoidance of smart ships. Finally, unlike the physical-model-based deblurring methods, the deblurring methods based on deep learning can directly restore the blurred images without using the physical model to estimate the blur kernel, but the process of image blurring can be modeled by the physical model. So, there should be a certain relevance between the two methods that achieve the same goal; however, there is no experiment or evidence to prove their relevance at present as far as we know.

In this paper, we propose SharpGAN in response to the above mentioned problems. The contribution can be summarized as follows:

1. In order to enhance the network’s ability to extract image features and maintain better real-time performance to adapt to the actual autonomous navigation of smart ships, we introduce the RFB-s module into the deblurring network for the first time. It combines the advantages of inception and dilated convolution as well as lightweight structure and is able to effectively extract image features by simulating the perceptual characteristics of human vision.
2. To make sure the restored images and the sharp images have more similar image features, we proposed a new feature loss based on image features at different levels. The feature loss is constructed using the feature maps of multiple convolutional layers of the convolutional neural networks (CNN), which makes full use of the powerful image feature extraction abilities of the CNN and can better guide the deblurring network to learn deblurring.
3. The experiments conducted on both GOPRO and Singapore Marine Dataset (SMD) datasets reveal that the SharpGAN can restore sharper images than the existing algorithms and maintain the best prediction efficiency. In addition, it can also significantly improve the object detection performance of the blurred real sea images.
4. We attempt to reveal the relationship between the SharpGAN and deblurring methods based on physical model through experiments, and our experimental results show that there is a certain relevance between the two methods.

## 2. Related Work

### 2.1. Image Deblurring

The process of image blurring can be represented by the following model

(1) $$I_{B}=I_{S}*K+N$$

where *I_B_* is the blurred image, *I_S_* is the latent sharp image, *K* is the blur kernel, and *N* is the noise. The latent sharp image *I_S_* is convolved with the blur kernel, and noise is added to produce the blurred image *I_B_*. According to whether the blur kernel is known or not, the methods of image deblurring can be divided into non-blind deblurring methods and blind deblurring methods.

Most of the early image deblurring methods are non-blind. These methods need to deconvolve the blurred image under the condition that the blur kernel is known to solve the restored image, such as the Lucy–Richardson algorithm [1,2], Wiener filter algorithm [17], and algorithm based on total variational model [18]. Compared with the non-blind deblurring method, the blind deblurring method can restore the blurred images under the condition that the blur kernel is unknown. Since Fergus et al. [3] successfully used the variational Bayesian method to achieve blind deblurring of images, many blind deblurring methods have been proposed successively. Shan et al. [4] used a piecewise function to fit the gradient distribution of the blurred image and then used an alternate iteration method to estimate the latent sharp image and blur kernel. Krishnan and Fergus [5] employed the normalized sparsity measure to estimate the blur kernel, and the application of the regularization term based on the image gradient improved the restoration effect of the blurred images. Xu et al. [7] proposed the method based on the unnatural L_0_ norm to estimate the blur kernel. The convolutional neural network and Markov theory were used by Sun et al. [10] to estimate the blur kernel at the patch level to remove non-uniform motion blur. Liu et al. [8] calculated the point spread function (PSF) based on angular velocity of the gyroscope and then combined the Richardson–Lucy (RL) algorithm to iteratively deblur the star image. The fully convolutional deep neural network was employed by Gong et al. [13] to estimate the motion flow of the blurred images, and then the method of [19] was employed to solve latent sharp images. Yan et al. [9] adopted the half quadratic splitting algorithm to iteratively solve the latent sharp image and blur kernel based on the light and dark channel theory. Li et al. [20] observed that natural images are full of self-repetitive structures and can be represented by similar patterns and proposed an improved sparse representation model for single image deblurring.

With the rapid development of deep learning in recent years, many end-to-end image deblurring methods have been proposed. Noroozi et al. [11] added the skip connection to the deblurring network, so that the network only needs to learn the residual between the blurred image and the sharp image, thereby reducing the difficulty of network learning. Nah et al. [12] used a large number of ResBlocks [15] to construct a multi-scale convolutional neural network, which gradually deblurred from the low-resolution image to final high-resolution image. Based on the generative adversarial network [21], Kupyn et al. [14] proposed an end-to-end image deblurring network (DeblurGAN), which adopted the PatchGAN [22], as the discriminator and the network was optimized by adversarial loss and content loss. Since then, Kupyn et al. [23] proposed a new deblurring method (DeblurGANv2) based on conditional generative adversarial network (C-GAN). DeblurGANv2 employed inceptionv2 as the backbone of the generator and combined the feature pyramid networks (FPN) to assist the network in extracting blurred image features.

### 2.2. Generative Adversarial Networks

Inspired by the idea of two-person zero-sum game in game theory, Goodfellow et al. [21] proposed the generative adversarial network (GAN), which mainly includes two architectures: the generator *G* and the discriminator *D*. Among them, the generator *G* uses noise data z (z obeys Gaussian distribution or other prior probability distributions) to generate the generated samples and makes the generated samples as real as possible to deceive the discriminator *D*. That is, the generator *G* tries to make the discriminator *D* think that the generated samples are real. The discriminator *D* improves its discrimination ability by continuously discriminating generated samples and real samples to prevent itself from being deceived by generator *G*, and the discrimination results (difference information between generated samples and real samples) are fed back to the generator *G* to make it generate more real samples. The training process of GAN is a game between the generator *G* and the discriminator *D*, and the loss function of the network can be expressed as:

(2) $$\underset{G}{\min}\underset{D}{\max}V(D,G)=E_{x\sim P_{data}}[\log(D(x))]+E_{x\sim P_{G}(x)}[\log(1-D(x))]$$

where *P_data_* is the data distribution of the real samples, and *P_G_* is the data distribution of the generated samples.

The proposal of GAN is extremely imaginative and creative, but its original network structure is not perfect, and there are a series of problems such as mode collapse and vanishing/exploding gradient. The root of these problems is that the loss function of GAN is to minimize the JS (Jensen–Shannon) divergence between the real samples and the generated samples. In high-dimensional space, using the JS divergence, it is often difficult to reflect the difference between the two data distributions, so it cannot provide effective gradient information for the network. In response to this problem, Arjovsky et al. [24] proposed Wasserstein GAN (WGAN), which adopted Wasserstein distance to replace the JS divergence in the loss function of original GAN and effectively reflected the difference between the real samples data distribution and the generated samples data distribution. The loss function of WGAN is expressed as follow:

(3) $$\underset{G}{\min}\underset{D\in D^{*}}{\max}V(D,G)=E_{x\sim p_{data}}[D(x)]-E_{x\sim p_{G}(x)}[D(x)]$$

where D* is the set of 1-Lipschitz functions. In order to make the discriminator meet the 1-Lipschitz continuity condition, Arjovsky et al. [24] employed the weight clipping method to limit the weight of the discriminator to the range of [−c, c], but the selection of c can easily lead to the vanishing/exploding gradient problem. For this reason, Gulrajani et al. [25] proposed WGAN with gradient penalty (WGAN-GP), which added the gradient penalty term

(4) $$E_{x}{\left[||\nabla_{x}D(x)||-1\right]}^{2}$$

to the loss function of WGAN. This made the training of GAN more stable and effectively solved the vanishing/exploding gradient problem of WGAN.

## 3. Proposed Method

Since the problem of image deblurring is highly ill-posed, the blurring effect of images is affected by factors such as the noise and the relative motion between the visual sensor and the shooting scene during the shooting process, which makes the restoration very complicated. The traditional methods obtain the latent sharp image by establishing physical models [1,2,3,4,5,6,7,8,9], but the restoration effect is highly dependent on the parameter settings in the model. So, they are difficult to adapt to the actual deblurring problem. There are also some methods that adopt CNN to estimate the blur kernel and motion flow. After that, the estimated blur information is used to deblur the image [10,13]. The efficiency of these non-end-to-end deblurring methods are low, and it takes more than ten seconds to process each image.

**Figure 1:**
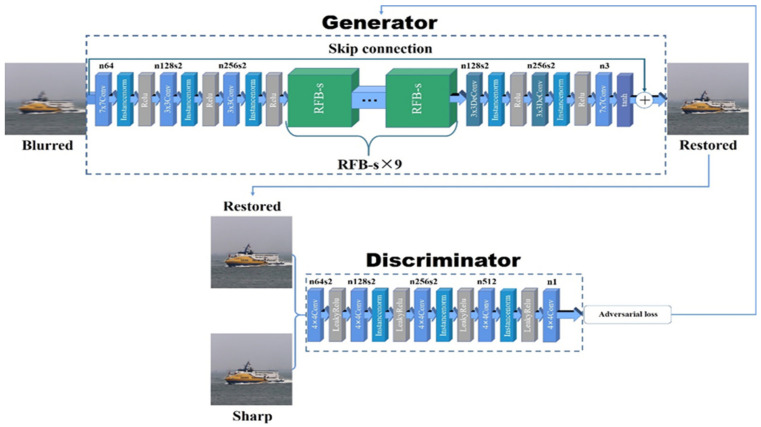
The architecture of the SharpGAN network. The generator of SharpGAN has 4 convolutional layers, 2 deconvolutional layers and 9 RFB-s modules, and the discriminator is similar to PatchGAN [22].

In this paper, we propose the SharpGAN based on the generative adversarial network, which includes two parts: generator and discriminator. The network structure of SharpGAN is shown in Figure 1. The generator uses the blurred image to directly generate the restored image and makes the restored image as sharp as possible to deceive the discriminator. It tries to make the discriminator think that the generated restored image is the sharp image. The discriminator prevents itself from being deceived by the generator through continuously discriminating the restored image and the sharp image in the high-dimensional space, and the difference information between them (discrimination result) is passed to the generator to guide it to generate the higher-quality restored image. The game between the generator and discriminator improves the deblurring performance of SharpGAN. In addition, the abstract difference between the blurred image and the sharp image is difficult to express through specific mathematical models or loss functions. However, as a generative model, GAN can effectively capture the feature differences between different data features and achieve effective end-to-end motion deblurring, which has unique advantages in deblurring tasks.

When the training of SharpGAN is completed, the generator has learned how to deblur under the guidance of the discriminator during the training process. So, in real application, we just need to deploy the model of the generator on the smart ship and input the blurred image to the generator to get the restored image. There is no need for the sharp image and discriminator.

### 3.1. Network Architecture

#### 3.1.1. Generator

**Figure 2:**
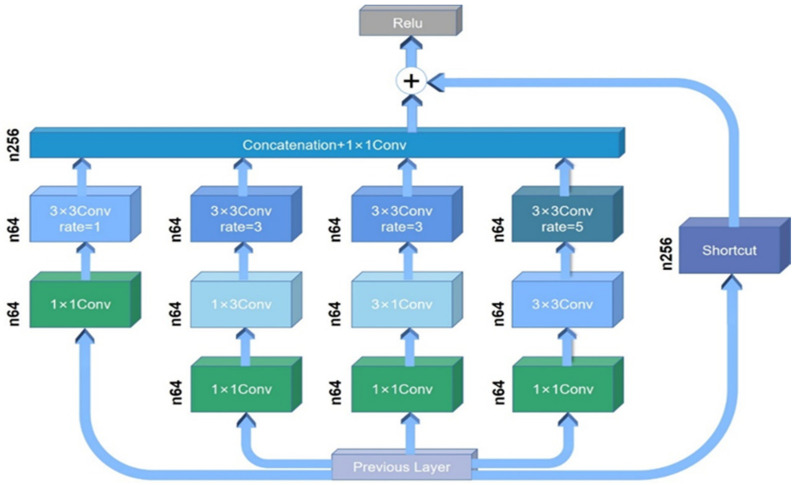
The architecture of the RFB-s module [26].

In the proposed SharpGAN, the generator uses the blurred image as the input to generate the latent sharp image, as in [14]. The front end of generator uses 3 convolutional layers to initially extract the blurred image features, and the back end of generator uses 2 deconvolutional layers and 1 convolutional layer to reconstruct the restored image with the same resolution as the input. The main difference with [14] is that in the middle part of the generator, the ResBlocks is replaced by 9 receptive field block net (RFBNet) modules [26], which makes the generator more powerful in image feature extraction. RFBNet has two structures: RFB and RFB-s. In order to make generator more lightweight and improve the real-time performance of deblurring, we chose the RFB-s module with fewer parameters as the part of the generator, and its network structure is shown in Figure 2.

The RFB-s module can be divided into 5 branches. Each branch first uses a 1 × 1 convolutional layer to reduce the dimension of the input data, so the network requires less calculation. Motivated by residual network’s (ResNet) skip connection [15], a branch called Shortcut is directly connected to the activation layer of the module. Meanwhile, inspired by the network structure of Inception [27,28,29], convolution kernels of different sizes are set in the other branches of the module. The advantages are that convolution kernels of different sizes can obtain different receptive fields, which are able to enhance the scale adaptability of the module to input samples and capture different levels of information in the image. At the end of the branch, the dilated convolution is employed. Compared with the ordinary pooling process, the dilated convolution expands the receptive field without reducing the image resolution, which is beneficial to enrich the image features extracted by the module [30]. After the dilated convolution processing, the four branches are spliced in the channel dimension and processed by 1 × 1 convolution, then merged with the data of another branch, and finally, output by the Relu activation layer.

In addition to the RFB-s modules, the generator network has 4 convolutional layers and 2 deconvolutional layers. Instance normalization is used to normalize the output results of all convolutional layers to speed up the network training process. Except for the last convolutional layer, which adopts tanh as the activation function, all other convolutional layers adopt Relu.

In this paper, referring to the method of [11], the global skip connection is employed in the generator, which adds the input of the network directly to the output. In this way, what the generator learns is the residual between the blurred images and the sharp images. It can be denoted:

(5) $$I_{S}=I_{B}+I_{R}$$

where *I_R_* is the residual between the latent sharp image *I_S_* and blurred image *I_B_*. The principle behind is that, since blurred images and sharp images have many similar image features in terms of color and style, compared to letting the network learn the mapping from blurred images to sharp images, only letting it learn the residual between them can reduce the difficulty of learning and make it converge faster during the training process.

#### 3.1.2. Discriminator

In the proposed network, the discriminator is responsible for discriminating sharp images and restored images. While continuously improving the discrimination ability, the discriminator also feeds back the discrimination results (adversarial loss) to the generator to guide the generator’s learning.

The discriminator in our network is similar to PatchGAN [22]. Different from the ordinary discriminator, PatchGAN divides the entire image into several patches, and the output discrimination result is a two-dimensional matrix. Each element in the matrix represents the discrimination result of the corresponding patch. Obviously, PatchGAN is able to guide the generator to perform deblurring learning more precisely than the ordinary discriminators.

### 3.2. Loss Function

In this paper, the loss function includes three components: adversarial loss, feature loss, and L2 loss.

#### 3.2.1. Adversarial Loss

Since the adversarial loss of the original GAN has many problems such as mode collapse and vanishing/exploding gradient, in order to avoid these problems and make the training process of SharpGAN more stable, the Wasserstein distance [24] with the gradient penalty [25] is used to represent the adversarial loss, which can be denoted by

(6) $$L_{adv}=\sum_{n=1}^{N}\left(D(I_{S})-D(G(I_{B}))\right)+\lambda_{\mathrm{GP}}\cdot E_{x}{\left[||\nabla_{x}D(x)||-1\right]}^{2}$$

where *N* is the number of images, *D* is the discriminator, *G* is the generator, and *G(I_B_)* is the restored image. The first item in Formula (6) represents the score of the sharp image and the restored image by the discriminator. In the training process, the discriminator *D* maximizes the adversarial loss to improve the score of the sharp image and reduces the score of the restored image, while the generator *G* minimizes the adversarial loss to improve the score of the restored image, as in [21]. That is, the confrontation between them improves the deblurring performance of the network. The second term of Formula (6) is the gradient penalty term, and *λ_GP_* is its weight constant.

#### 3.2.2. Feature Loss

In DeblurGAN [14], the author employed the content loss, which used the features of conv_3-3 layer extracted from VGG19 network, to improve the feature similarity between the restored images and the sharp images. Considering that convolutional neural network has strong image feature extraction abilities, the image feature levels extracted from the shallow and deep layers of the network are different. The shallow layers of the network are good at extracting simple color, edge, and texture feature, and the deep layers of the network are good at extracting high-level semantic feature [31]. Therefore, only using a certain layer of the deep convolutional neural network to construct the loss function of the feature does not make full use of the feature extraction ability of the convolutional neural network.

**Figure 3:**
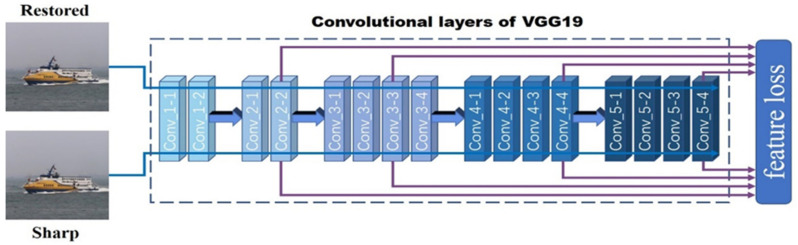
The feature loss fuses the different layers of features extracted from the VGG19 network.

In this paper, we proposed the feature loss based on deep convolutional neural network, which adopts the feature maps of the shallow and deep layers to fuse different levels of image features to help the network better learn motion deblurring. This is another major innovation of the SharpGAN. In Figure 3, we take the VGG19 network as an example to introduce the implementation process of the feature loss.

The VGG19 network has a total of 16 convolutional layers. When calculating the feature loss, the sharp image and restored image are input to the VGG19 network, respectively, to obtain their feature maps at different layers, and the mean square error of these featuremaps is calculated to reflect the feature difference between the sharp image and the restored image. In order to effectively cover the image features of different levels extracted by the network, conv_2-2, conv_3-3, conv_4-4, and conv_5-4 layers are selected to construct feature loss.

The features loss function is defined by:

(7) $$L_{F_{i,j}}=\frac{1}{W_{i,j}H_{i,j}}\sum_{x=1}^{W_{i,j}}\sum_{y=1}^{H_{i,j}}\left(\varphi_{i,j}{\left(I_{S}\right)}_{x,y}-\varphi_{i,j}{\left(G(I_{B})\right)}_{x,y}\right)\overset{2}{}$$

where *φ_i,j_* is the feature map obtained by the j-th convolution (after activation) before the i-th maxpooling layer within the VGG19 network, and *W_i,j_* and *H_i,j_* are the width and height of the feature maps.

#### 3.2.3. L2 Loss

In order to minimize the pixel error between the restored image and the sharp image, we add the L2 loss to the loss function, as in [12]. The L2 loss function is as follow:

(8) $$L_{2}=\sum_{n=1}^{N}{\left(I_{S}-G(I_{B})\right)}^{2}$$

Combining adversarial loss, feature loss, and L2 loss can get the total loss of our network:

(9) $$\underset{\min G}{\arg}\underset{\max D}{\arg}L(D,G)=L_{adv}+\lambda_{F}\cdot L_{F}-\lambda_{2}\cdot L_{2}$$

where *λ_F_* is the weight constant of feature loss, and *λ_2_* is the weight constant of L2 loss.

## 4. Discussion of Relevance between the Two Deblurring Methods

There are two main categories of deblurring methods in the literature as mentioned above: deep-learning-based and physical-model-based. The former is mostly based on generative adversarial network (GAN) or convolutional neural network (CNN) to achieve end-to-end deblurring, and the process of deblurring does not need to estimate the blur kernel [11,12,14]. The latter usually can be divided into three parts: estimation of blur kernel, deconvolution and removing noise, and iterative optimization, as in [4]. The theoretical basis of these methods is Formula (1), and their deblurring process can be expressed by the Formula (10), which is the inverse process of Formula (1).

(10) $$I_{S}=(I_{B}-N)*K^{-1}$$

Compared with the deblurring methods based on the physical model, we find that the deblurring method based on deep learning (SharpGAN) is highly similar to them. We can also divide the network structure of SharpGAN into three parts. The first part is the RFB-s modules and the convolutional layers before them in the generator. The second part is the deconvolutional layers in the generator and the convolutional layers after them. The third part is the discriminator. The first part can be seen as extracting the features of the blurred image through continuous convolution to estimate the blur kernel K. The second part uses the blur kernel K to deconvolve the blurred image and remove noise. The third part is responsible for identifying the effect of the restored image and iteratively guiding the deblurring of the first and second parts (generator). Therefore, from a macro point of view, we believe that the two methods are essentially related, and the process of deblurring are both based on Formula (10).

It is very interesting to reveal the relationship between these two categories of methods. In this paper, we take the SharpGAN as an example to build a bridge between these two methods from couple perspectives: the mapping from the sharp (restored) image to the blurred image and the residual between the sharp (restored) image and the blurred image.

### 4.1. Relevance between Blurred Images

Assuming that the essence of SharpGAN is Formula (10). Then, the blurred image *I_B_G_*, which is obtained by implementing Formula (1) (i.e., the inverse process of Formula (10)) on the restored image *I_G_* of SharpGAN, as shown in Formula (11), should be highly similar to the original blurred image *I_B_*.

(11) $$I_{B\_G}=I_{G}*K+N$$

### 4.2. Relevance between Residuals

Considering that SharpGAN learns the residuals of the blurred image *I_B_* and the sharp image *I_S_*, we also attempt to prove the relevance between the SharpGAN and deblurring method based on the physical model from the perspective of the residuals.

It can be derived from Formulas (1) and (5) that

(12) $$I_{R\_S}=I_{S}-I_{B}=I_{S}-(I_{S}*K+N)$$

where *I_R_S_* is the residual between the sharp image and the blurred image.

We also assume that the essence of SharpGAN is Formula (10). Furthermore, the residuals *I_R_G_*, which is obtained by replacing the sharp image *I_S_* in Formula (12) with the restored image *I_G_* of SharpGAN, as shown in Formula (13), should also be highly similar to *I_R_S_*.

(13) $$I_{R\_G}=I_{G}-(I_{G}*K+N)$$

Due to the complexity of deep-learning-based method, its deblurring process is difficult to visualize. So, we attempt to prove that the essence of deblurring method based on deep learning (SharpGAN) is Formula (10) through experiments and then prove that the two methods are related in Section 6.3.

## 5. Training

In this paper, the GOPRO [12] and Singapore Marine Dataset (SMD) datasets [32] are exploited to reveal the performance of the proposed SharpGAN.

The GOPRO dataset [12], which is widely used in the community of deblurring, employs high frame rate cameras to record videos of different dynamic scenes. The average value of multiple consecutive frames in the video is adopted to simulate real blurred images. There are 2013 and 1111 blurred and sharp image pairs in the training set and the test set, respectively.

The SMD dataset [32] is taken in the sea area near Singapore port and divided into two parts: on-shore video and on-board video. We randomly select 3000 images from the SMD dataset, of which 1500 are employed as the training set, 500 are employed as the test set, and 1000 are employed as the dataset of relevance experiment. Then, the motion blur is added to the sharp SMD dataset based on the Formula (1). For smart ships, motion blur is caused by the swaying motion of the ship in the navigation. This will blur the entire image captured by the vision sensor, which is a global motion blur. Due to the influence of wind, waves, and currents, smart ships will undergo heave, trim, roll, pitch, and other motions during navigation. These motions are random, and the direction and distance of the motions are variable. To better fit the actual motion blur problem, we set multiple blur sizes from 16 to 40 pixels, and the blur angle is randomly sampled from 0 to 360° with uniform distribution, as in [33,34]. Finally, we add Gaussian white noise with standard deviation σ = 0.01 to each blurred image. After the above process, the total images in the training set, test set, and dataset of relevance experiment are 3000, 1000, and 2000, respectively.

The training process has a total of 500 epochs for each dataset. The learning rate is set to 10^−4^ in the first 250 epochs and linearly decays to 0 in the last 250 epochs. The batch size in the training stage is set to 1. In order to balance the proportion of each loss in the total loss, we set the weight constant *λ_GP_* of gradient penalty term to 10, the weight constant *λ_2_* of L2 loss to 10^6^, and the weight constant *λ_F_* of feature loss to 1. Through experiments, we determined that the conv_2-2, conv_3-3, conv_4-4, and conv_5-4 layers of the VGG19 network [16] accounted for 0.2, 0.4, 0.2, and 0.2 in the feature loss. So, the final feature loss in the experiment is denoted as:

(14) $$L_{F}=0.2L_{F_{2,2}}+0.4L_{F_{3,3}}+0.2L_{F_{4,4}}+0.2L_{F_{5,4}}$$

In the training stage, we proposed a multi-scale training method. In each step of the training process, we randomly select a scale from 256 × 256, 384 × 384, 512 × 512, and 640 × 640 to crop the images used for network training to adapt to different scales of motion blur. After that, the random mirror or geometrical flip is employed to the cropped images to augment the training set.

In order to more clearly reveal the improvement of network performance, we have successively employed the RFBNet module, the feature loss with multi-level image features, and the multi-scale training method to the network. The specific information of each model in the training process is shown in Table 1, where the training of Baseline Model and Baseline Model+ used image patches with a size of 512 × 512.

## 6. Experimental Results

We implement our model with TensorFlow, and all the following experiments are performed on a single NVIDIA GeForce GTX 2080Ti GPU and 4 Inter Core i3-10100 CPUs. The peak signal to noise ratio (PSNR) and structural similarity (SSIM) are selected as the evaluation criteria of the experiments. The two criteria, respectively, measure the quality of image restoration in terms of pixel error and structural similarity (brightness, contrast, and structure). The better the quality of the restored image, the higher the two criteria.

### 6.1. GOPRO Dataset

In the field of motion deblurring, the GOPRO dataset [12] is one of the most popular datasets. In order to verify the deblurring performance of SharpGAN, we first compared it with the existing algorithms on the GOPRO dataset. The experimental results are shown in Table 2. It can be seen from Table 2, SharpGAN has obvious advantages in PSNR and real-time performance of deblurring and has a slight gap in SSIM compared with the method of [12].

**Figure 4:**
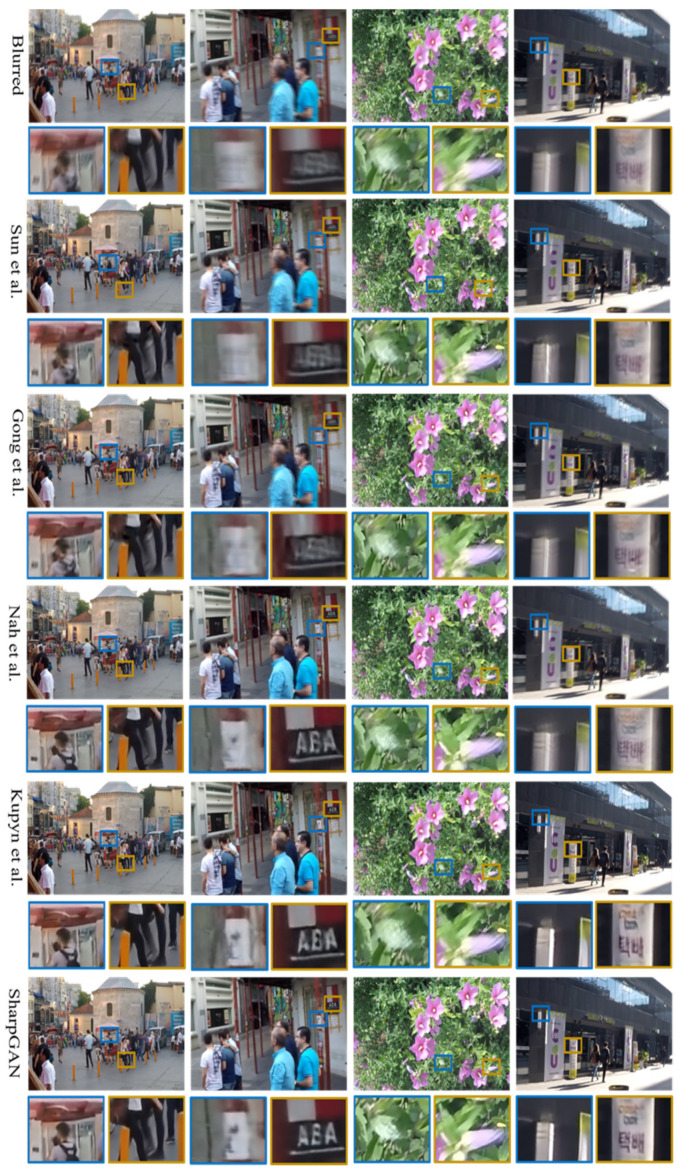
The deblurring results of GOPRO dataset [12]. From top to bottom: blurred images, the results of Sun et al. [10], Gong et al. [13], Nah et al. [12], Kupyn et al. [23], and the results of SharpGAN.

Figure 4 shows that SharpGAN has the excellent deblurring effect in complex dynamic scenes. It avoids unnatural visual effects such as edge distortion and excessive sharpening. Compared with the contrast methods, it has a better ability to restore the texture details of the objects in the blurred images, such as the outline of pedestrian legs, billboards on the street, and the edges of petals. Besides, it can also significantly reduce the artifacts of the restored images. So, the restored images of SharpGAN are more real and natural.

### 6.2. SMD Dataset

To verify the effectiveness of SharpGAN in removing motion blur of real sea images, we evaluated it on the SMD dataset [32]. The quantitative results of different methods are reported in Table 3.

In Table 3, the PSNR of Baseline Model is higher than that of all the methods in the comparison. By introducing the feature loss with multi-level image features and multi-scale training method to the network, both PSNR and SSIM are gradually improved. Though the SSIM of SharpGAN has a very small gap with the method of [12], it produces the best results on the PSNR criterion. While maintaining the high-quality deblurring, the proposed method also significantly shortens the deblurring time. It reserves more time for the subsequent detection algorithm to detect obstacles, which is of great help to the autonomous navigation of smart ships.

**Figure 5:**
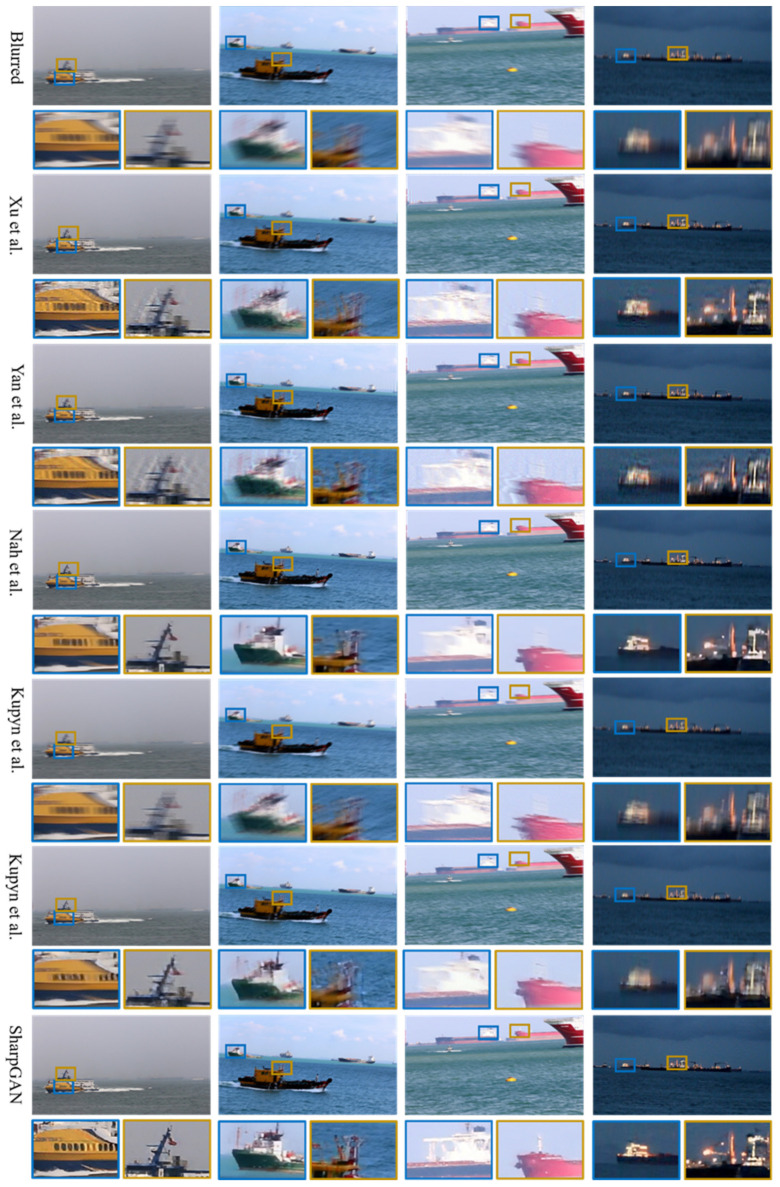
The deblurring results of SMD dataset [32]. From top to bottom: blurred images, results of Xu et al. [7], Yan et al. [9], Nah et al. [12], Kupyn et al. [14], Kupyn et al. [23], and the results of SharpGAN.

Figure 5 displays some visual examples of the SMD dataset [32]. Compared to the other methods, SharpGAN can more effectively improve the clarity of the blurred image and better restore the complex edge and texture of the ships’ mast, hull, and superstructure. The restored images retain the original detail information well, and there is no obvious ringing phenomenon.

Since object detection is an important part of the visual perception of smart ships, we adopt the pretrained Single Shot MultiBox Detector (SSD) ship object detection model of [35] to detect the blurred images, sharp images, and restored images of different methods to verify the improvement of the object detection effect of SharpGAN.

Table 4 lists the quantitative detection results of different methods. SharpGAN is superior to its competitors in precision, recall, and F1 score, and there is a slight gap compared to the results of sharp images.

**Figure 6:**
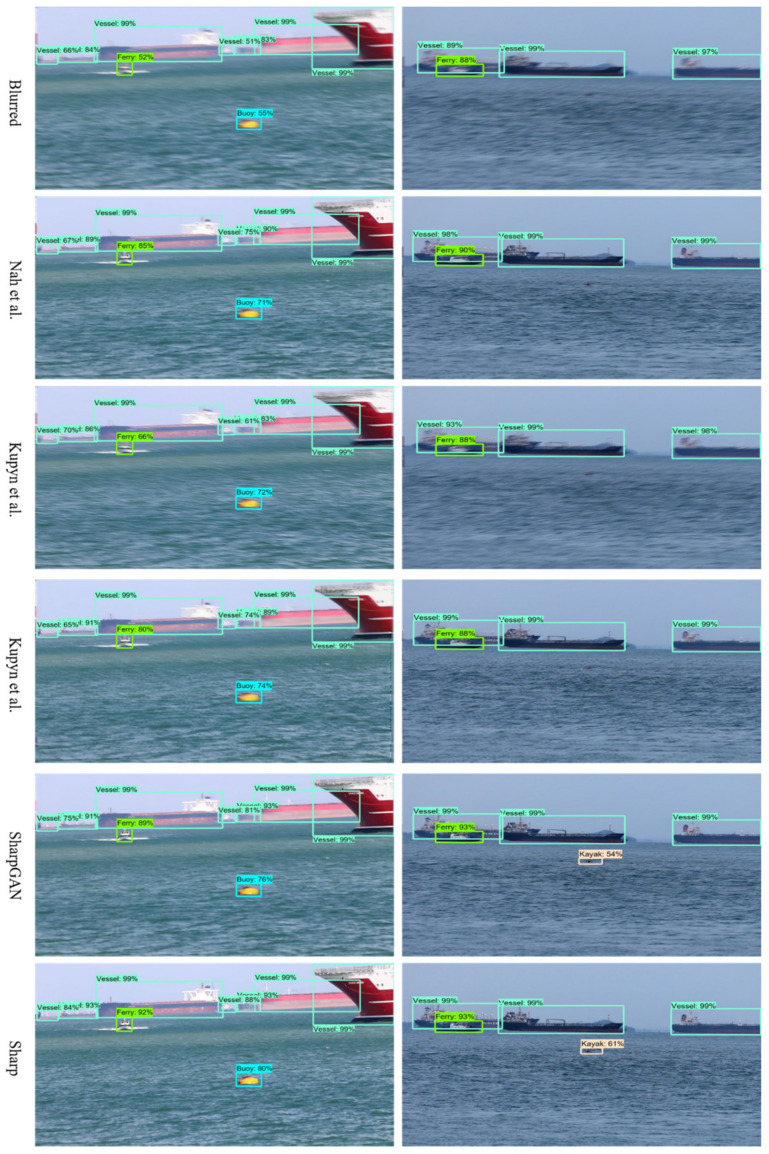
The object detection results of the SMD dataset [32]. From top to bottom: results of blurred images, Nah et al. [12], Kupyn et al. [14], Kupyn et al. [23], SharpGAN, and the results of sharp images.

Figure 6 shows that SharpGAN produces the best object detection results. It can better reconstruct the contour features of the objects compared to all contrast methods, especially small objects, so that the detection results of the restored images are able to approach to that of the sharp images. These qualitative and quantitative results explain that SharpGAN is of great significance to the visual perception and navigation safety of smart ships.

### 6.3. Relevance between the Two Methods

Based on the discussion in Section 4, we attempt to reveal the relationship between the deep-learning-based method and the physical-model-based method. We at first generate the blurred images *I_B_* using 1000 sharp images *I_S_* in the SMD dataset [32] with Formula (1), while preserving the corresponding blur kernels *K* and noise *N*. The specific technical information of the blur kernel *K* and noise *N* is as stated in Section 5. Secondly, we input the blurred images *I_B_* into SharpGAN trained with a different number of epochs to obtain a series of restored images *I_G_*. The epochs we selected are from 0 to 500 with the interval of 50. Finally, we used the above *I_S_*, *I_G_*, *K*, and *N* to generate corresponding *I_R_S_*, *I_R_G_*, and *I_B_G_* based on Formulas (11)–(13). After that, we calculated the SSIM and MSE of *I_R_S_* and *I_R_G_*, and SSIM and PSNR of *I_B_* and *I_B_G_*, respectively.

**Figure 7:**
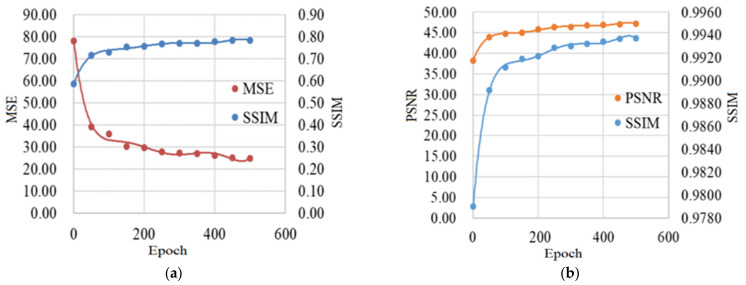
Relevance experiment results. (**a**) The SSIM and MSE of *I_R_S_* and *I_R_G_*; (**b**) the SSIM and PSNR of *I_B_* and *I_B_G_*.

It can be seen from Figure 7a that when the training epoch of SharpGAN increases, the SSIM of *I_R_S_* and *I_R_G_* also increases; in contrast, the MSE gradually decreases. Figure 7b also shows that as SharpGAN’s training epoch increases, the PSNR and SSIM of *I_B_* and *I_B_G_* also increase. The experimental results show that the essence of SharpGAN can be expressed as the Formula (10) to some extent. So, combined with the discussion in Section 4, it is reasonable to believe that the deblurring method based on deep learning (SharpGAN) has a high correlation with the method based on the physical model.

## 7. Conclusions

In this paper, we proposed SharpGAN, a new deblurring method based on the generative adversarial network, and attempted to reveal the relevance between the deblurring method based on physical model and SharpGAN through experiments. The RFBNet module [26] was introduced into the deblurring network, and the feature loss was proposed to fuse multi-level image features. The experimental results showed that SharpGAN had outstanding real-time performance while ensuring high-quality deblurring. Compared with the existing methods, it had obvious advantages in both qualitative and quantitative aspects and can also improve the object detection effect of blurred real sea images.

By using the real sea image dataset and combining a variety of blur parameters to construct the experimental training set, the generalization ability of SharpGAN has been obviously improved, so that it is possible to maintain a certain deblurring effect under unknown scenes. However, the training set cannot cover all scenarios, i.e., the data cannot be obtained in advance in all navigation scenarios. Therefore, in future work, we will try to adopt self-supervised or unsupervised learning methods to better fit the autonomous navigation of smart ships.

## Figures and Tables

**Table 1:** Components of each model.

Method	Components
Baseline Model	RFBNet + content loss based on the conv_3-3 layer of the VGG19 network
Baseline Model+	RFBNet + feature loss with multi-level image features
SharpGAN	RFBNet + feature loss with multi-level image features + multi-scale training

**Table 2:** The experimental results of different methods on the GOPRO dataset.

Method	Main Property	PSNR	SSIM	Time	Hardware
Whyte et al. [6]	Based on geometric properties of cameras	23.80	0.836	30 min	4 CPUs
Sun et al. [10]	Estimating blur kernel based on CNN	24.64	0.843	18 min	4 CPUs
Gong et al. [13]	Estimating motion flow based on CNN	26.06	0.863	18 min	4 CPUs
Nah et al. [12]	Based on multi-scale CNN	29.23	0.916	1.13 s	Single GPU
Kupyn et al. [23]	Based on GAN and FPN	27.63	0.855	0.18 s	Single GPU
SharpGAN	Based on GAN, RFBNet, feature loss, and multi-scale training	29.62	0.897	0.17 s	Single GPU

**Table 3:** The experimental results of different methods on the SMD dataset.

Method	Main Property	PSNR	SSIM	Time	Hardware
Xu et al. [7]	Estimating blur kernel based on unnatural L_0_ sparse representation.	28.59	0.792	18.68 s	4 CPUs
Yan et al. [9]	Based on light and dark channel theory	27.24	0.637	10 min	4 CPUs
Nah et al. [12]	Based on multi-scale CNN	30.88	0.856	2.11 s	Single GPU
Kupyn et al. [14]	Based on GAN	25.75	0.596	0.57 s	Single GPU
Kupyn et al. [23]	Based on GAN and FPN	29.05	0.799	0.39 s	Single GPU
Baseline Model	Based on GAN and RFBNet	31.71	0.832	0.37 s	Single GPU
Baseline Model+	Based on GAN, RFBNet, and feature loss	31.84	0.835	0.37 s	Single GPU
SharpGAN	Based on GAN, RFBNet, feature loss, and multi-scale training	31.90	0.837	0.37 s	Single GPU

**Table 4:** Quantitative detection results of SMD dataset.

Method	Precision	Recall	F1 Score
Blurred images	0.453	0.339	0.388
Nah et al. [12]	0.590	0.429	0.497
Kupyn et al. [14]	0.533	0.372	0.438
Kupyn et al. [23]	0.544	0.403	0.463
SharpGAN	0.622	0.445	0.519
Sharp images	0.625	0.445	0.520
